# The effect of oral essential amino acids on incretin hormone production in youth and ageing

**DOI:** 10.1002/edm2.85

**Published:** 2019-07-26

**Authors:** Haitham Abdulla, Joseph J. Bass, Tanner Stokes, Stefan H. M. Gorissen, Chris McGlory, Bethan E. Phillips, Stuart M. Phillips, Kenneth Smith, Iskandar Idris, Philip J. Atherton

**Affiliations:** ^1^ MRC‐ARUK Centre for Musculoskeletal Ageing Research and NIHR BRC, School of Medicine University of Nottingham Derby UK; ^2^ Diabetes and Endocrinology Centre University Hospitals Birmingham NHS Foundation Trust, Heartlands Hospital Birmingham UK; ^3^ Department of Physical Education and Sport Sciences University of Limerick Limerick UK; ^4^ Department of Kinesiology McMaster University Hamilton Ontario Canada; ^5^ Department of Endocrinology and Diabetes University Hospitals Derby and Burton NHS Foundation Trust Derby UK

**Keywords:** ageing, essential amino acids, incretin effect, incretins, insulin

## Abstract

**Background:**

The effect of substantive doses of essential amino acids (EAA) on incretin and insulin production, and the impact of age upon this effect, is ill‐defined.

**Methods:**

A 15‐g oral EAA drink was administered to young (N = 8; 26 ± 4.4 years) and older (N = 8; 69 ± 3.8 years) healthy volunteers. Another group of younger volunteers (N = 9; 21 ± 1.9 years) was given IV infusions to achieve equivalent plasma amino acids (AA) profiles. Plasma AA, insulin, glucagon‐like peptide‐1 (GLP‐1) and glucose‐dependent insulinotropic peptide (GIP) were quantified over 2 hours.

**Results:**

In younger recruits, EAA‐induced rapid insulinaemia and aminoacidaemia with total amino acids(AA), EAA and branched chain amino acids (BCAA) matched between oral and IV groups. Insulin peaked at 39 ± 29 pmol L^−1^ at 30 minutes following oral feeding compared to 22 ± 9 pmol L^−1^ at 60 minutes following IV feeding (*P*: NS). EAA peaked at 3395 μmol L^−1^ at 45 minutes during IV infusion compared to 2892 μmol L^−1^ following oral intake (Feeding effect: *P* < 0.0001. Oral vs IV feeding: *P*: NS). There was an 11% greater increase in insulin levels in the 120 minutes duration of the study in response to oral EAA as opposed to IV EAA. GIP increased following oral EAA (452 pmol L^−1^ vs 232 pmol L^−1^, *P* < 0.05). Age did not impact insulin or incretins production.

**Conclusion:**

Postprandial rises in EAA levels lead to rapid insulinaemia which is higher with oral compared with IV EAA, that is attributed more to GIP and unaffected by age. This finding supports EAA, on their own or as part of high‐protein meal, as nutritive therapeutics in impaired glycaemia and ageing.

## 1 INTRODUCTION

Oral glucose has been shown to stimulate greater insulin release compared to a comparable glucose challenge given intravenously^1^ (measured as the difference between insulin attributed to oral vs IV glucose load). This difference is attributed to the secretion of incretin hormones such as glucagon‐like peptide‐1 (GLP‐1) and glucose‐dependent insulinotropic peptide (GIP)—known as the “incretin effect,” produced from the L and K cells respectively, and accounts for up to 65% of the amount of insulin secreted following oral glucose ingestion.^1^, ^2^ Orally ingested lipids have also been shown to induce the incretin effect due to postprandial elevations in both GLP‐1 and GIP.^3^

The extent to which these incretin hormones are produced in response to oral intake of dietary proteins and amino acids (AA) remains poorly defined. Nonetheless, it has been shown that consumption of a mixture of oral amino acids (AA) stimulates GLP‐1 and GIP secretion.^4^ Moreover, in the only study assessing the true incretin effects of AA, Lindgren et al^5^ reported an incretin effect of ~25% attributed mainly to rises in GIP (rather than GLP‐1) following a small dose (6.5 g) of mixed (ie, essential AA [EAA] and non‐EAA) vs comparable I.V AA administration. Since the estimated daily protein requirement for a 70 kg adult is 0.8 g kg^−1^ d^−1^,^6^ studies to investigate the impact of more typical AA meal intake of >10 g of AA (~20 g protein) on incretin hormones production is important. Moreover, determining the effects of EAA vs NEAA on insulin production is important in relation to determining the key AA drivers of the incretin hormone productions.

Ageing of the gut and gut‐brain axis is a burgeoning area of research. Ageing is associated with reduced energy intake, protein malnutrition (in some instances) and impaired anabolic responsiveness to protein foods^7^, ^8^, ^9^, ^10^, ^11^, ^12^; likely contributing to deleterious shifts in body composition—engendering sarcopenia, age‐related insulin resistance and metabolic ill‐health.^13^ These factors are facilitated by age‐related changes in gastrointestinal (GI) physiology in response to mixed meal^14^, ^15^, ^16^—which could play an important role in nutrient deficiency and predisposition to age‐related sarcopenia. Yet the effects of EAA alone on incretin and insulin hormone production, and in relation to age, remains poorly defined. Crucially, while oral lipid and glucose intake did not lead to differences in either L and K cells responses in younger and older people,^17^ older individuals exhibited higher levels of GIP (not GLP‐1) compared to younger subjects after oral protein consumption.^18^

In the postabsorptive state, mobilization of NEAA (mainly glutamate and alanine) from skeletal muscle represent the main mechanism via which splanchnic tissues are provided with fuel at the expense of negative muscle protein balance.^19^ During the fed state, splanchnic tissues rely on enteral extraction of glutamine and alanine with most EAA appearing in the systemic circulation.^20^ Therefore, EAA, due to their lesser influence on splanchnic tissue gluconeogenesis, could be regarded as an important nutraceutical strategy for the regulation of muscle and glucose metabolism. In addition, this later fact makes EAA a suitable testing mixture to allow more accurate estimation of protein‐derived incretin secretion. Also, since EAA are the primary drivers of muscle protein synthesis^21^, ^22^, ^23^ while incretin hormones are critical to glucose metabolism, studies investigating the effects of oral vs intravenous EAA in relation to incretin hormones are relevant to promoting healthy ageing. The aim of this study was thus to investigate: (a) the extent oral EAA could induce increases in incretin and insulin hormone production, and (b) if ageing impacts upon these postulated EAA‐induced effects.

## 2 SUBJECTS AND METHODS

### 2.1 Study design

The study was approved by The University of Nottingham Ethics Committee and Hamilton Integrated Research Ethics Board and was conducted in line with the Declaration of Helsinki and registered at https://clinicaltrials.gov/ (NCT02370745). A total of 25 healthy volunteers were recruited to participate. Seventeen healthy young males (18‐30 years) and eight healthy older males (65‐75 years) were recruited via local advertisement in the two sites of the study (University of Nottingham—Derby—UK and McMaster University—Hamilton—Canada). All volunteers gave informed consent. All participants had no history of diabetes, liver disease or gastrointestinal abnormalities. They were assigned to three groups. (a) Young oral (Y.O) group (Nottingham site): eight young aged to have oral EAA beverage. (b) Young intravenous (Y.IV) group (Hamilton site): nine young aged to have equivalent intravenous EAA. (c) Old oral (O.O) group (Nottingham site): eight older aged to have the same oral EAA beverage as the Y.O group. Please see Table 1 for the subjects' characteristics.

**Table 1:** Characteristics of study participants

Parameter (mean ± SD)	Y.O (n = 8)	Y.IV (n = 9)	O.O (n = 8)	*P*
Age (y)	26 ± 4.4	22 ± 1.9	69 ± 3.8	****
Height (m)	1.80 ± 0.1	1.79 ± 0.03	1.70 ± 0.1	*
Body mass (kg)	81.0 ± 11.8	78.1 ± 8.8	75.8 ± 5.1	NS
BMI (kg m^−2^)	25.0 ± 2.8	24.4 ± 2.8	25.5 ± 1.9	NS
FPG	4.3 ± 0.9	4.4 ± 0.6	4.9 ± 1.0	NS

Abbreviations: FPG, fasting plasma glucose; NS, not significant; O.O, older group consumed oral EAA; Y.IV, young group given intravenous EAA; Y.O, young group consumed oral EAA.

**** *P* < 0.0001 O.O vs Y.O and O.O vs Y.IV.

* *P* < 0.05 O.O vs Y.O and O.O vs Y.IV.

**Figure 1:**
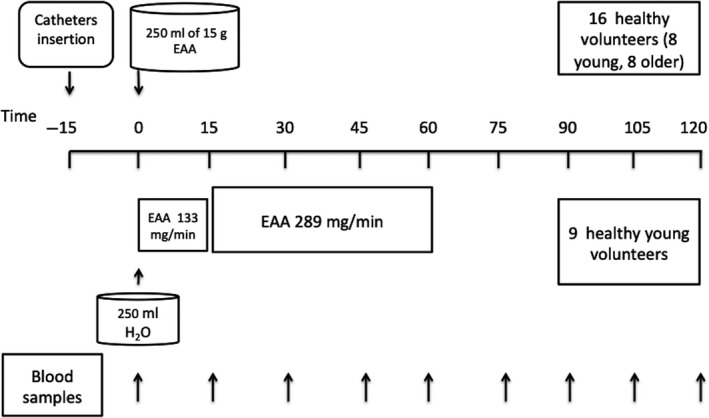
Schematic representation of the study protocol. Eight young and eight older male volunteers orally ingested a total of 15 g essential amino acids (EAA). Another group of nine young male volunteers received 15 g of intravenous EAA infusion

All groups reported to the study sites following overnight fasting of 8‐14 hours. An 18‐20G cannula was inserted into the antecubital vein for blood sampling at baseline (*t* = 0 minute) and at 15, 30, 45, 60, 75, 90, 105 and 120 minutes post consumption of either 15 g of oral EAA solution (YO and OO groups) or following initiation of intravenous 15 g of equivalent EAA infusion (group Y.IV.). After baseline sampling, volunteers (YO and OO groups) were asked to consume a pre‐prepared oral EAA beverage. The solution was prepared the day before in 250 mL of aqueous solution, kept refrigerated overnight and warmed to room temperature in the morning of the study. The stability of the EAA components of the solution following overnight stay was tested beforehand. The composition of the EAA beverage was l‐histidine: 1.21 g; l‐isoleucine: 1.73 g; l‐leucine: 3.59 g; l‐lysine: 3.07 g; l‐methionine: 0.95 g; l‐phenylalanine: 0.91 g; l‐threonine: 1.13 g; l‐tryptophan: 0.48 g and l‐valine 1.86 g.^24^ Following the last sample (120 minutes), the cannula was removed and volunteers were offered a snack. Participants were kept at the study site for 30 minutes observation before being allowed to leave. The group of eight young males receiving an intravenous equivalent of 15 g EAA (Stepping Hill Hospital pharmacy, Stockport, UK) received two cannulae; one for blood sampling as described above and the second for infusion of EAA. The EAA infusate contained equivalent amounts of EAA to the oral beverage. EAA were infused for a total of 60 minutes. Following sampling for baseline measurements, EAA infusion was started at a rate of 133 mg min^−1^ for 15 minutes after which the rate of infusion was increased to 289 mg min^−1^. These rates were calculated aiming to deliver 13% of total EAA infusate in the first 15 minutes and 87% over the remaining 45 minutes guided by previous reports.^5^ With the start of infusion, volunteers were given 250 mL H_2_O to compensate for stomach distention. At the end of infusion (60 minutes), blood sampling continued every 15 minutes for another hour to complete a total of 120 minutes of sampling. At the end of the study, cannulae were removed and volunteers were offered a snack. They remained on site for monitoring as described above. See Figure 1 for the schematic representation of the study protocol.

### 2.2 Measurements and calculations

#### 2.2.1 Measurement of plasma AA and glucose concentrations

For measurement of AA concentrations, internal standards were added to plasma samples before the addition of urease solution and incubation at room temperature for 20 minutes. Samples were then de‐proteinized with ice‐cold ethanol for 20 minutes at 4°C, before centrifugation at 13 000 *g*. The plasma AA containing supernatant was then decanted into boiling tubes and evaporated at 90°C under N_2_. Dried AA were vortex mixed in 0.5 mol L^−1^ HCl and lipids extracted in Ethyl Acetate, before evaporation to dryness. AA were derivatized through the addition of equal volumes of acetonitrile (ACN) and N‐tert‐Butyldimethylsilyl‐N‐methyltrifluoroacetamide (MTBSTFA) and heated to 90°C for 60 minutes. Samples were allowed to cool before transfer to autosampler vials. Glucose concentrations were determined similarly through the inclusion of an internal standard before the samples were de‐proteinized with ice‐cold ethanol and evaporated to dryness. Accurate analysis of glucose by GC‐MS is routine to our laboratories as final batch analyses. Samples were collected in sodium fluoride tubes, stored immediately on ice, centrifuged in small batches throughout the study, then aliquoted and stored at −80°C until analysis by GC‐MS.^25^ Glucose samples were derivatized through the addition of oxime reagent to dried samples, vortex mixed and heated at 85°C for 30 minutes, before the addition of N,O‐Bis(trimethylsilyl)trifluoroacetamide (BSTFA) and further heated at 85°C for 30 minutes. Both AA and glucose concentrations were quantified against a standard curve of known concentrations using GC‐MS.

#### 2.2.2 Measurement of insulin and gut hormones concentrations

Commercial ELISA kits (Milliplex Map kit; EMS Millipore) were used to determine insulin and gut hormone concentrations. Blood samples for these analysis were collected in P800 tubes, that specifically stabilize GLP‐1 and GIP upon collection. The kit was the Milliplex Map Kit—Human metabolic hormone magnetic bead panel (Cat #HMHEMAG‐34K) measured on a Luminex‐Magpix. This is commercially validated kit that has been certified to have a linear range for GLP‐1 of 2.7‐2000 mg mL^−1^ and for GIP 1.4‐1000 pg mL^−1^, both shown to have a recovery of 103% from serum matrix. The analytes were insulin, C‐peptide, GIP total, GLP‐1 total and glucagon. The incretin effect was calculated as the difference between insulin responses after oral and intravenous EAA stimulation, relative to the response seen after oral EAA ingestion.

### 2.3 Power of calculation and statistical analysis

The sample size was prospectively determined with a power of calculation and taking a population (inter‐ and intraindividual) variance of 15% (based on previous laboratory data on insulin and AA concentrations on young and old individuals) and CV of laboratory techniques also of 15% to detect differences in feeding modes with 80% confidence at the 5% significance level. The analysis was conducted using Prism 7 (GraphPad). Data are presented as mean ± SD. Normality of distribution was tested using D'Agostino and Pearson Omnibus normality tests. Comparison between two measures made at times before and after feeding or same time point between two groups were made via repeated measures 2‐way ANOVA with Bonferroni post‐test analysis to determine significance. Change from baseline was calculated as the ratio between time point measurement and calculated average baseline value. The net incremental area under the response curve (AUC) was calculated for each individual separately and presented as a two‐group comparison.

## 3 RESULTS

### 3.1 Plasma AA concentrations

**Figure 2:**
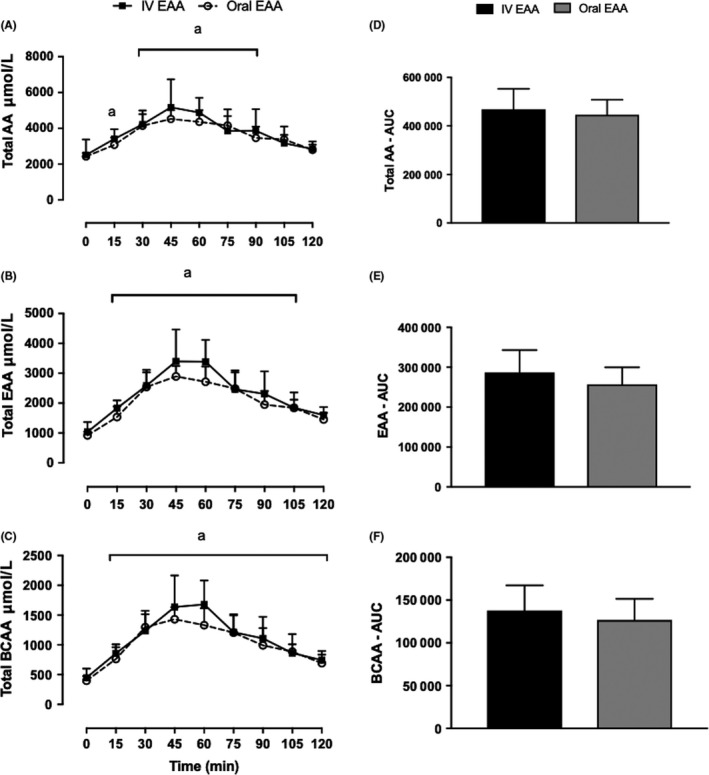
Plasma total AA (A), EAA (B) and BCAA (C) concentrations after oral vs IV EAA, demonstrated with AUC in (D), (E) and (F), respectively. ^a^Greater than respective baseline (*P* < 0.05). Main effect of feeding, *P* < 0.0001; effect of feeding type, NS; Interaction, *P* < 0.05. Values are expressed as mean ± SD. Measured by 2‐way ANOVA. AA, amino acids; BCAA, branched‐chain amino acids; EAA, essential amino acids

For both Y.O and Y.IV groups, concentrations were matched with no significant difference between the two groups at baseline for total AA, EAA and branched‐chain amino acid (BCAA; Figure 2—results evaluated via RM 2‐way ANOVA). Total AA, EAA and BCAA were significantly higher than correspondent baseline values at 15 minutes (*P* < 0.05) in both groups with exception of total AA in Y.O group, which started to become significantly higher at 30 minutes. Total AA reached a peak of 5170 ± 1654 μmol L^−1^ (1‐fold rise from baseline) in Y.IV group compared to a peak of 4523 ± 487 μmol L^−1^ (0.9 fold rise) in Y.O group at 45 minutes (*P*: NS). EAA peaked at 3395 ± 1072 μmol L^−1^ (2.3 fold rise from baseline) at 45 minutes during IV infusion compared to 2891 ± 350 μmol L^−1^ (2.1 fold rise from baseline) following oral intake (*P*: NS). BCAA reached a peak of 1678 ± 401 μmol L^−1^ (2.7‐fold rise from baseline) in Y.IV group compared to 1330 ± 284 μmol L^−1^ (2.6‐fold rise from baseline) in the Y.O group at 60 minutes (*P*: NS). Non‐essential amino acid (NEAA) concentrations remained unchanged from baseline in both groups throughout the 120‐minute duration of the study. Total AA concentrations returned to baseline at 120 minutes in Y.O group, as opposed to 105 minutes in the Y.IV group. Total EAA and BCAA were back to baseline at 105 and 120 minutes, respectively.

There was no significant difference between individual EAA concentrations at all time points with the exception of threonine and histidine in the Y.IV at 45 and 60. However, change from baseline was not different for threonine at these time points.

### 3.2 Glucose, insulin, C‐peptide and glucagon

**Figure 3:**
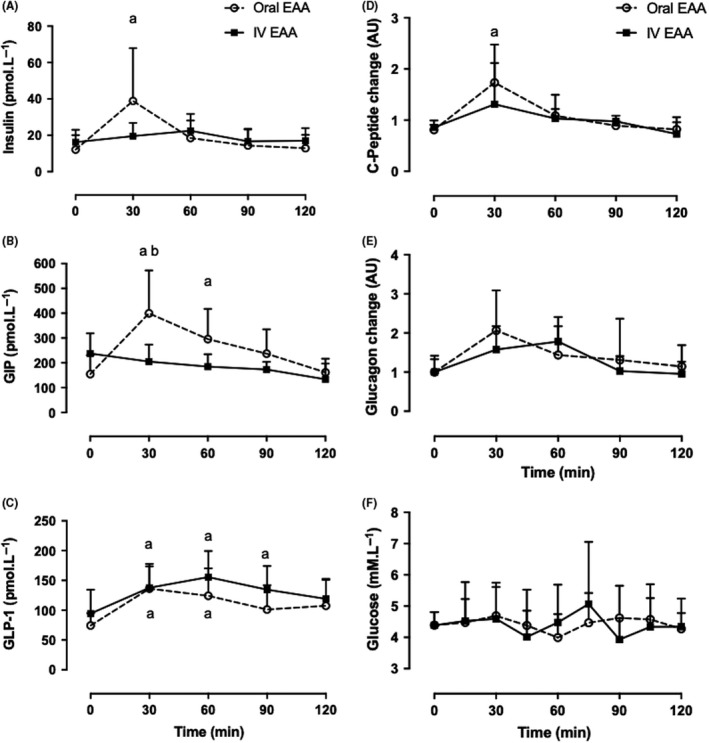
Oral vs IV EAA. Plasma insulin, incretin hormone concentrations, C‐peptide, glucagon and glucose. Insulin (A), GIP (B) and GLP‐1 (C). C‐peptide and glucagon changes from baseline are plotted in (D) and (E) respectively. Glucose concentrations are shown in (F). ^a^Greater than respective baseline (*P* < 0.05). ^b^Between feeding types (*P* < 0.05). Values are expressed as mean ± SD. C‐peptide and glucagon data are displayed as ratio from baseline mean. Measured by 2‐way ANOVA. EAA, essential amino acids; GIP, glucose‐dependent insulinotropic peptide; GLP‐1, glucagon‐like peptide‐1

Analyses were conducted in Y.O and Y.IV groups. There was no difference in glucose concentration between the two young groups at baseline. Insulin peaked at 39 ± 29 pmol L^−1^ at 30 minutes in the Y.O group, which was significantly elevated compared to baseline (2.3‐fold rise from baseline, *P* < 0.05). In comparison, insulin concentrations in Y.IV group peaked at 22 ± 9 pmol L^−1^ at 60 minutes (0.4‐fold rise from baseline) and overall remained not different from baseline throughout the study. The difference in insulin rise between the two groups (the incretin effect) was 45% calculated as the difference between AUC for the first 60 minutes and was 11% calculated as AUC difference for the entire 120 minutes duration of the study. C‐peptide change at 30 minutes from baseline was 1.7 ± 0.7 folds (*P* < 0.5 vs baseline) in the Y.O group compared to 1.3 ± 0.8 folds change from baseline (*P*: NS against baseline) in the Y.IV group with no significant difference between the two groups at this time point. This rise corresponded to that seen in insulin concentrations. Glucagon concentrations rose from baseline peaking at 30 minutes following oral EAA intake (2 fold rise, *P*: NS) and at 60 minutes following IV EAA administration (1.8 fold rise, *P*: NS; Figure 3).

### 3.3 Incretin hormones

Total GLP‐1 concentrations rose significantly compared to baseline at 30 minutes (0.8‐fold rise from baseline) in the Y.O group (136 ± 41 vs 74 ± 20 pmol L^−1^, *P* < 0.05). In comparison, GLP‐1 concentrations in the Y.IV group stayed unchanged through the 120‐minute duration of the study. No difference was observed between feeding types in GLP‐1 concentrations. Total GIP concentrations rose significantly following oral consumption to peak at 30 minutes (1.6 fold rise from baseline) while remaining unchanged throughout the study in the Y.IV group. This rise was significantly different between the two groups (399 ± 173 vs 205 ± 68 pmol L^−1^, *P* < 0.05; Figure 3).

### 3.4 Ageing and AA acid profile

**Figure 4:**
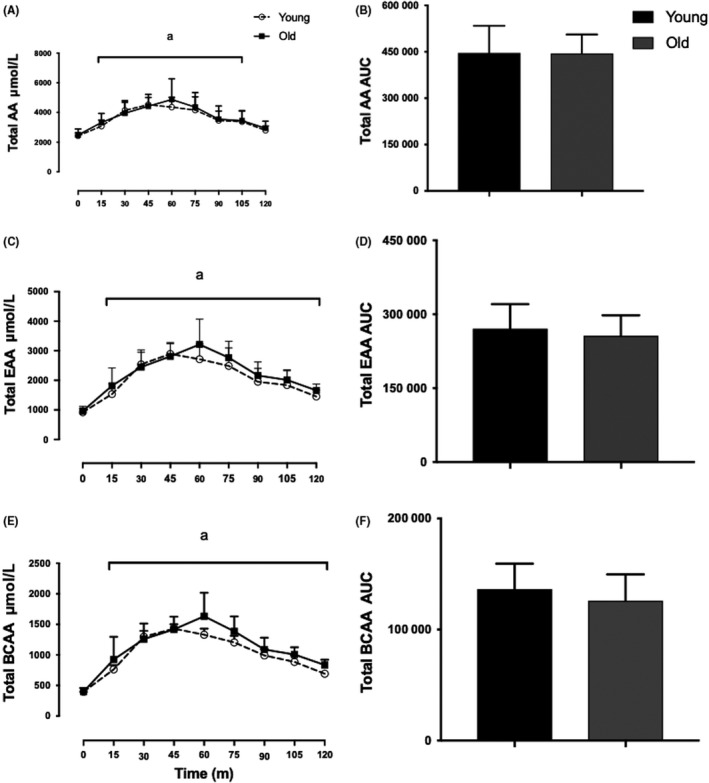
Young vs Old plasma AA profile. Plasma concentrations of total AA (A), total EAA (C) and BCAA (E) plotted alongside AUC in (B), (D) and (F), respectively. ^a^Greater than respective baseline (*P* < 0.05). Main effects of feeding, *P* < 0.0001, effects of age, NS, Interaction, NS. Values are expressed as mean ± SD. Measured by 2‐way ANOVA

Following oral consumption of EAA beverage in the Y.O and O.O groups, AA profiles were similar between the two groups at all time points. At 15 minutes, total AA, EAA and BCAA were significantly elevated compared to baseline. Total AA, EAA and BCAA peaked at 60 minutes in both groups (Y.O vs O.O: 4357 ± 659 vs 4868 ± 1402 μmol L^−1^, 2716 ± 513 vs 3214 ± 858 μmol L^−1^ and 1330 ± 284 vs 1632 ± 386 μmol L^−1^, respectively, *P*: NS)—Figure 4.

### 3.5 Ageing and incretin hormone release

**Figure 5:**
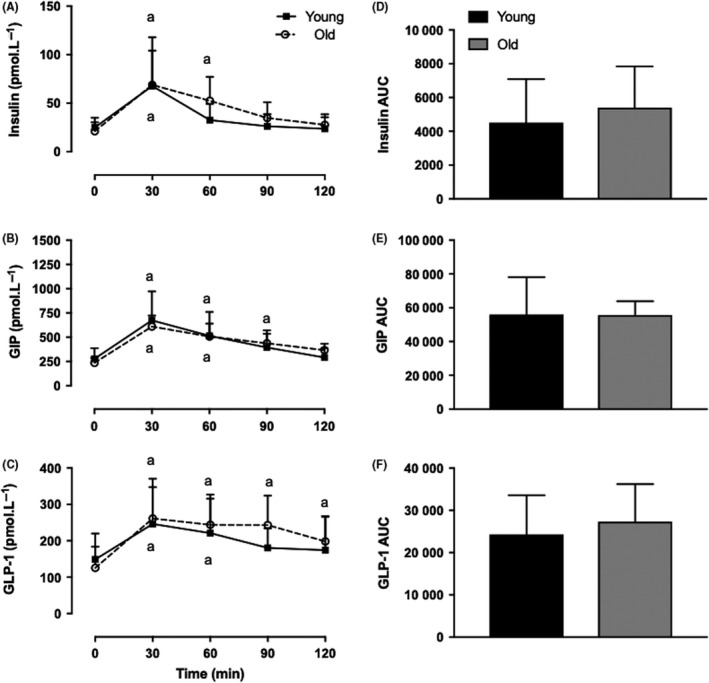
Young vs Old plasma insulin and incretin hormone release following oral consumption of 15 g EAA. Insulin (A), GIP (B) and GLP‐1 (C) concentrations, demonstrated with AUC in (D), (E) and (F), respectively. ^a^Greater than respective baseline (*P* < 0.05). Main effects of feeding, *P* < 0.0001; effects of age, NS; Interaction, NS. Values are expressed as mean ± SD. Measured by 2‐way ANOVA. EAA, essential amino acids; GIP, glucose‐dependent insulinotropic peptide; GLP‐1, glucagon‐like peptide‐1

Insulin, GLP‐1 and GIP rose similarly between Y.O and O.O groups in response to oral consumption of EAA. All hormones peaked at 30 minutes (67 ± 50 vs 68 ± 35 pmol L^−1^, 246 ± 101 vs 261 ± 109 pmol L^−1^ and 594 ± 264 vs 538 ± 99 pmol L^−1^; Y.O vs O.O for insulin, GLP‐1 and GIP, respectively, *P*: NS) with a slower return to baseline in the O.O group (Figure 5).

## 4 DISCUSSION

In this study, we report that 15 g of orally consumed EAA increased plasma insulin concentrations which are higher following oral compared with IV EAA challenge. We are not aware of any other study that examined such an effect in relation to EAA. This rise and fall in insulin was matched by a significant rise followed by a fall in GIP. GLP‐1 also had risen significantly from baseline but the fact that insulin levels fell despite the continued elevation of GLP‐1 concentration points to lack of causal effect. The difference in insulin concentration with oral vs IV is likely to be attributed the incretin hormones (and likely GIP) was ~11% calculated as total AUC over 120 minutes. This effect is EAA‐related since no gluconeogenic source was infused in our study. It is also important to highlight the use of a dose (15 g EAA) relating to main meal protein consumption and thus any reported results could be easily extrapolated to translate clinically if an equivalent EAA beverage is provided instead. Finally, our studies have demonstrated that this effect of incretin hormone production (at least in response to EAA) does not appear to be affected by the process of physiological ageing.

Incretin and insulin responses to protein are established.^26^ Nonetheless, excluding a single study by Lindgren et al,^5^ the effect of AA on incretin hormones release remains poorly defined. Moreover, this previous study employed AA mixtures, containing both essential and non‐essential AA. This could, in theory, be problematic when it comes to assessing the “incretin effects” as some NEAA are known to be gluconeogenic precursors, namely alanine (kidney) and glutamine (liver and intestine). Indeed, glutamine is the main source of energy for the gut, especially the small intestine, through the gluconeogenic production of glucose.^27^ Therefore, any quantification of an “incretin effect” would be difficult to ascribe solely to AA. The use of EAA in this study represents a novel approach to avoid this potential confounder while focusing upon the impacts of diet‐derived (essential) AA components of dietary protein.

Stimulation of GIP but not GLP‐1 by oral protein ingestion has been reported previously.^28^ Enteroendocrine cells are scattered along the length of the gut making it the largest endocrine organ in the body. In our study, GLP‐1 in both Y.O and Y.IV rose comparably from baseline. The fact that there was no change in insulin concentration in the Y.IV group makes insulin rise in the Y.O group highly unlikely contributed to by GLP‐1. Most likely GLP‐1 concentrations achieved in both modalities of feeding are not high enough to stimulate insulin secretion. This lack of GLP‐1‐mediated insulin secretion following protein intake might be related to absorption sites in the bowel; K cells are predominantly present in the duodenum and ileum. By the time AA reaches the jejunum, where L cells are more predominant, there might be very little substrate left to stimulate L cells. Another explanation could be that L cells are not as sensitive to AA compared to K cells. This does not exclude a small contributory effect for GLP‐1 on insulin secretion from neuronal stimulation within the gut. In contrast to this, data from mice and cell cultures reported evidence of an AA‐stimulated GLP‐1 release, namely glutamine, glycine, alanine and phenylalanine.^29^

Our results show that providing 15 g of EAA stimulates glucagon production, although the rise overall was not significantly different from baseline. Glucagon stimulation was previously reported following mixed AA and whey protein.^30^, ^31^ It was suggested that AA and glucagon regulate each other in a feedback loop that involves α‐cells and hepatocytes.^32^ In comparison to AA, orally or intravenously delivered glucose suppresses glucagon secretion, which is more pronounced with the latter mode of delivery.^2^ Incretin hormones have a contrasting effect on glucagon. While GIP stimulates glucagon secretion from pancreatic α‐cells, GLP‐1 inhibits its release. Further, pancreatic α‐cells are known to have a very small number of GLP‐1 receptors compared to GIP receptors, which are ubiquitously expressed within α‐cells.^33^ Compared to glucose consumption, the contrasting glucagon response seen in this study is likely a response to rises in EAA concentration. Incretin release as a result of oral consumption seems to have no added effect on glucagon release.

The effect of incretin hormones on insulin production is crucial in alleviating postprandial hyperglycaemia in normal individuals. In individuals with type 2 diabetes where this effect is impaired,^1^ strategies that potentiate insulin secretion are vital in controlling postprandial blood glucose excursions. Further, postprandial glycaemia has proven to be a better predictor for HbA1c compared to fasting blood glucose.^34^ Therefore, although our study was performed in normoglycaemic individuals, we believe that optimising AA mixtures could represent a feeding strategy for individuals with impaired glycaemia. For example, a previous study has shown that consumption of whey protein prior to a standard breakfast was associated with a significant reduction in postprandial glucose, driven by increased in post prandial GLP‐1 and insulin concentration compared with control.^35^ The amount of 15 g of EAA in our study also ensures that a sufficient meal portion is delivered per meal in a daily AA requirement of 24‐56 g of protein per day for a 70 kg adult. In addition to this antiglycaemic property showed in our study, EAA have the advantage of superior “anabolic profile” compared to other dietary protein mixes.^36^

We acknowledge that the lack of cross‐over comparison could be a potential source for variability between the two young groups. We also acknowledge the fact that the physiological process of digestion and absorption is subject to both intra and inter‐individual variations and that a cross‐over design may have been more powerful. However, we think the well‐matched plasma appearance of EAA across the groups and the greater capture of biological variance are validatory and study strengths.

We did not detect age‐related differences in gut hormone responses to EAA. This finding suggests that, despite adverse reports on ageing related gut physiology,^37^, ^38^, ^39^ the function of gut incretin secretion, at least in response to EAA, remains largely intact with ageing.^18^ Nonetheless, older age did appear to be related to a slower return (albeit not significant) of incretin and insulin to baseline following oral consumption (Y.O and O.O groups); we suspect this may be due to increased rates of clearance of EAA in the young. In contrast, AA profiles were also similar between both age groups indicating that digestion, absorption and splanchnic extraction of EAA is minimally affected by the ageing process. Therefore, nutritional strategies aimed at improving health performance and skeletal muscle health in the older population should focus on the quality of diet and appetite enhancement. Future directions could aim at further scrutinising the mechanisms by which EAA stimulate L cells and whether this could be translated into more potent stimulation (ie, higher GLP‐1 concentration) as a result of EAA mixture consumption.

## 5 CONCLUSION

We have shown that EAA are able to induce rises in incretins and insulin hormones. There was a demonstrable differential effect of EAA on insulin production when given orally and matched with an intravenous equivalent. Our results also suggest that an EAA‐induced effect is delivered through hormonal rises likely related to GIP. Our results add to those from a series of studies previously proved that the “incretin effect” exists following consumption of glucose, mixed AA and lipid beverage. In particular relation to AA‐induced incretin hormone production, we suggest using EAA gives more accurate estimate in this regards. Our results suggest that the process of physiological ageing does not adversely affect EAA‐induced incretin hormone release. Hence (although it was not directly estimated in this study), we predict that associated incretin effect in elderly population is likely to remain intact. Further research is needed to inform clinically or nutritionally meaningful intervention especially that could elucidate the mechanisms via which L and K cells sense EAA in the gut and EAA‐mediated hormones release in both youth and ageing.

## CONFLICT OF INTEREST

The authors declare no conflict of interest in relation to this manuscript.

## AUTHORS' CONTRIBUTIONS

P.J.A, I.I, K.S, B.E.P, H.A conceptualized the study. H.A, T.S and S.H.M.G performed clinical studies. J.J.B performed sample and statistical analysis. H.A drafted the initial manuscript, which was then reviewed and edited by all authors. All authors contributed to interpretation of results and approved the final version of the manuscript.

## ETHICAL APPROVAL

The study was performed according to the Declaration of Helsinki and was approved by The University of Nottingham Ethics Committee and Hamilton Integrated Research Ethics Board. All participants gave written informed consent following full explanation of the rationale for and conduct of the study, in addition to the procedures involved.

## Data Availability

The data that support the findings of this study are available from the corresponding author (PJA) upon reasonable request.
